# Everybody's Page

**Published:** 1892-05-28

**Authors:** 


### Everybody's Page.

Few people will be found to disagree with the policy of abandoning cumulative penalties for disobeying the vaccination laws, however, strong their views upon the necessity of compulsory vaccination may be. It is not easy to conceive of anything more likely to render the Acts so unpopular as to make them practically of no effect than such penalties.

---

A story is told in the *Medical Record* of how a hotel proprietor was hoist with his own petard by a doctor. The doctor, who arrived at the hotel late in the evening, at once went to bed. When looking over his bill, before leaving, he noticed he was charged for supper on the night of his arrival. His objection to the charge on the ground that he had had no supper was met by the clerk saying "but you were here and might have had it." The doctor at once made out a bill for medical services and presented it to the clerk, who replied he was not aware the doctor had been called on for medical service. "Oh, no," said the doctor, "but I was here, and I might have been." No further demand was made for the questioned item.

---

An American physician complained recently to a fellow practitioner, that he had great difficulty in procuring his fee from fathers of new born babes. His friend found a remedy for this state of affairs. On attending a case shortly afterwards and being asked if it would be quite as convenient were he to be paid his fee in a week's time, he promptly replied, "Quite, for I never lose any money on obstetrical cases." "Indeed," asked the parent. "Well," said the doctor, "it is becoming a well-established superstition based upon facts, that parents who allow their infant boy to start in life with a debt hanging over his head are sure to have a ne'er-do-well son, and the girl in such a predicament is sure to marry a pauper." The feelings of the anxious mother could not bear this awful strain, and the fee was duly paid.

The holiday season will soon be upon us, and people are already beginning to ask themselves and their friends "Where shall we go, and how?" There are few pleasanter ways of enjoying oneself than by a walking tour, which has many advantages. In a walk numerous points of interest and beauty can be seen which are missed when travelling in a train. There is, or need be, no hurry, and time is your own, which it certainly is not when you have to bustle after trains. The one great essential for a walking tour is comfortable boots. It should be remembered that in walking for any length of time the foot increases by nearly one-eighth of its normal size, therefore, boots should not be tight. Those who have tender feet will find the frequent soaking of the feet in alum baths for some days before they undertake their trip will have a beneficial and hardening effect.

---

Fashion, it seems, is not the mere blind caprice which some people have considered it to be, and there is a method in its madness and apparent impulses. According to Mr. Philip Newman the successive phases of human attire have observed a regular law of development from the primitive fig leaf of the Adamite period to the warmer, if more cumbersome attire of the present day. The toga of Julius Cæsar is therefore a connecting link between the leaf and the frock coat, and that illustrious Roman would have been in advance of his time, though distinctly original, had he invaded Great Britain in a silk hat and frock coat; but the moral effect of such apparel on the ancient Briton might have saved him many a fight. Just as the frock coat may be traced back to the toga, the modern silk hat might be traced to that peculiar coal scuttle with ventilating holes depicted in the illustrated histories of our youth as worn by the Crusader, who no doubt found it extremely useful for cooking purposes when campaigning, especially if there were any chestnuts near at hand.

